# Melatonin Treatment Reduces Oxidative Damage and Normalizes Plasma Pro-Inflammatory Cytokines in Patients Suffering from Charcot-Marie-Tooth Neuropathy: A Pilot Study in Three Children

**DOI:** 10.3390/molecules22101728

**Published:** 2017-10-14

**Authors:** Mariam Chahbouni, María del Señor López, Antonio Molina-Carballo, Tomás de Haro, Antonio Muñoz-Hoyos, Marisol Fernández-Ortiz, Ana Guerra-Librero, Darío Acuña-Castroviejo

**Affiliations:** 1 Departamento de Fisiología, Facultad de Medicina, Instituto de Biotecnología, Centro de Investigación Biomédica, Parque Tecnológico de Ciencias de la Salud, Universidad de Granada, 18016-Granada, Spain; abtyo@hotmail.com (M.C.); sol92@correo.ugr.es (M.F.-O.); ana.guerralibrero@gmail.com (A.G.-L.); 2 CIBERfes, Ibs.Granada, and UGC de Laboratorios Clínicos, Complejo Hospitalario de Granada, 18016-Granada, Spain; msenor.lopez.sspa@juntadeandalucia.es (M.d.S.L.); tomas.haro.sspa@juntadeandalucia.es (T.d.H.); 3 Unidad de Gestión Clínica de Pediatría, Hospital Universitario San Cecilio, 18012-Granada, Spain; amolinac@ugr.es (A.M.-C.); amunoz@ugr.es (A.M.-H.)

**Keywords:** antioxidant, cytokines, inflammation, melatonin therapy, oxidative stress, axonal neuropathy

## Abstract

Charcot-Marie-Tooth neuropathy (CMT) is a motor and sensory neuropathy comprising a heterogeneous group of inherited diseases. The CMT1A phenotype is predominant in the 70% of CMT patients, with nerve conduction velocity reduction and hypertrophic demyelination. These patients have elevated oxidative stress and chronic inflammation. Currently, there is no effective cure for CMT; herein, we investigated whether melatonin treatment may reduce the inflammatory and oxidative damage in CMT1A patients. Three patients, aged 8–10 years, were treated with melatonin (60 mg at 21:00 h plus 10 mg at 09:00 h), and plasma levels of lipid peroxidation (LPO), nitrites (NOx), IL-1β, IL-2, IL-6, TNF-α, INF-γ, oxidized to reduced glutathione (GSSG/GSH) ratio, and the activities of superoxide dismutase (SOD), glutathione-S transferase (GST), glutathione peroxidase (GPx), and reductase (GRd), were determined in erythrocytes at 3 and 6 months of treatment. Healthy age- and sex-matched subjects were used as controls. The results showed increased activities of SOD, GST, GPx, and GRd in CMT1A patients, which were reduced at 3 and 6 months of treatment. The GSSG/GSH ratio significantly increased in the patients, returning to control values after melatonin treatment. The inflammatory process was confirmed by the elevation of all proinflammatory cytokines measured, which were also normalized by melatonin. LPO and NOx, which also were elevated in the patients, were normalized by melatonin. The results document beneficial effects of the use of melatonin in CMT1A patients to reduce the hyperoxidative and inflammatory condition, which may correlate with a reduction of the degenerative process.

## 1. Introduction

Charcot-Marie-Tooth disease (CMT), also known as Charcot-Marie-Tooth neuropathy, hereditary motor and sensory neuropathy, and peroneal muscular atrophy, is a motor and sensory neuropathy comprised of a large heterogeneous group of inherited diseases [1,2,3]. The CMT clinical phenotype is one of the most common inherited neuromuscular disorders in humans, with an estimated prevalence of one case per 2500 individuals [4]. CMT was initially classified into two types; CMT1, in which nerve conduction velocities (NCVs) are reduced together with hypertrophic demyelination, is predominant in the 70% of CMT patients, and CMT2, with normal NCVs and axonopathy [1,5]. CMT is monogenic and more than 80 genes have been identified in relation with this disease [6]. The duplication of the gene coding for the peripheral myelin protein 22 on chromosome 17p11.2, is responsible for the CMT1A phenotype cursing with demyelinating and slow NCVs [7]. There is no effective cure for CMT, and only controlling the progress of the disease, physical therapy, and orthopedic treatment, are available to date. Early diagnosis can facilitate the CMT patients to modify their lifestyles to minimize the damage to the nerves to delay or prevent disability.

Besides clinical signs, CMT courses with the existence of increased acute, subacute, or chronic inflammation [8,9,10,11,12] and oxidative stress, with elevated production of reactive oxygen species (ROS). The hyperoxidative status in CMT patients depends on an increase in the glutathione-S-transferases (GSTs), a reduction of the glutathione (GSH) pool leading to enhanced oxidized glutathione (GSSG)/GSH ratio, and mitochondrial dysfunction [13,14]. Experimental studies in rats and mice demonstrate an association of peripheral neuropathy with a reduction of the antioxidant status with low levels of GSH, and of glutathione peroxidase (GPx) and reductase (GRd) activities, and an increased lipid peroxidation [15]. The excess of ROS induces the activation of NF-κB and NF-κB-dependent pro-inflammatory cytokines including IL-1b in CMT patients [16]. Evidences for macrophage-dependent inflammatory activation and myelin disruption has been shown in a murine model of CMT1A [17]. Therefore, chronic oxidative damage and inflammation, which exceed the ability of muscle regeneration [18], constitute an important pathogenic mechanism accelerating the progressive loss of muscle fibers in CMT patients.

This hyperoxidative status and inflammation suggest that antioxidant and/or anti-inflammatory therapy may have beneficial effects in CMT. In addition to its chronobiotic activity [19], melatonin is an important antioxidant with anti-inflammatory properties [20,21,22,23,24], Moreover, the capacity of melatonin to improve mitochondrial function and reduce the formation of ROS [25] and restore the GSH pool [26], yields further protective support to the damaged muscle. In this regard, experimental models of sepsis and aging provide strong evidence for the protective efficacy of melatonin against ROS and inflammation-induced skeletal and cardiac muscle impairment [27,28,29,30]. These properties of melatonin have been also evaluated in children suffering from Duchenne muscular dystrophy, showing significant improvements in the oxidative stress and inflammatory status of these patients [31,32].

Collectively, the data support trophic effects of melatonin on skeletal muscle [33,34,35]. With these considerations, we evaluated whether melatonin administration could reduce the oxidative stress and inflammation in CMT patients. We analyzed in these patients the intracellular oxidative stress status of erythrocytes before and after melatonin therapy, looking for a beneficial effect of melatonin in reducing the hyperoxidative status of these patients.

## 2. Results

### 2.1. Melatonin Reduces SOD and GST Activities

**Figure 1:**
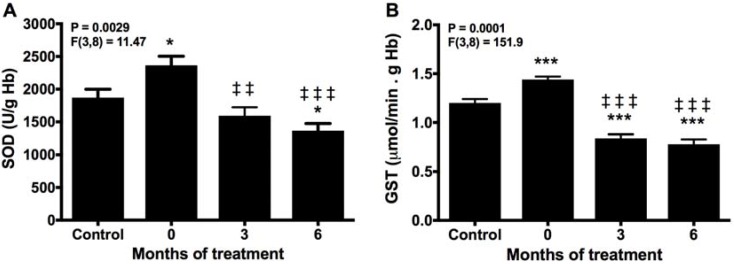
Erythrocyte activity of superoxide dismutase (SOD) (**A**) and glutathione-S transferase (GST) (**B**) before (0) and after 3 and 6 months of treatment with melatonin, compared with healthy controls. * *p* < 0.05, and *** *p* < 0.001 vs. control; ‡‡ *p* < 0.01, and ‡‡‡ *p* < 0.001 vs. 0.

The activity of SOD, the important enzyme in the antioxidant endogenous defense system, was higher in the patients before melatonin treatment than in healthy controls (Figure 1A). After 3 months of melatonin therapy, SOD activity declined to below controls, and it was further reduced at the end of the treatment. Comparable changes were found for GST activity, which was increased in the patients before starting the therapy, reduced below controls at 3 months of treatment; the GST activity remained at this level to the end of the study, i.e., 3 months later (Figure 1B).

### 2.2. Melatonin Recovery of the Normal Turnover of the Glutathione Cycle

**Figure 2:**
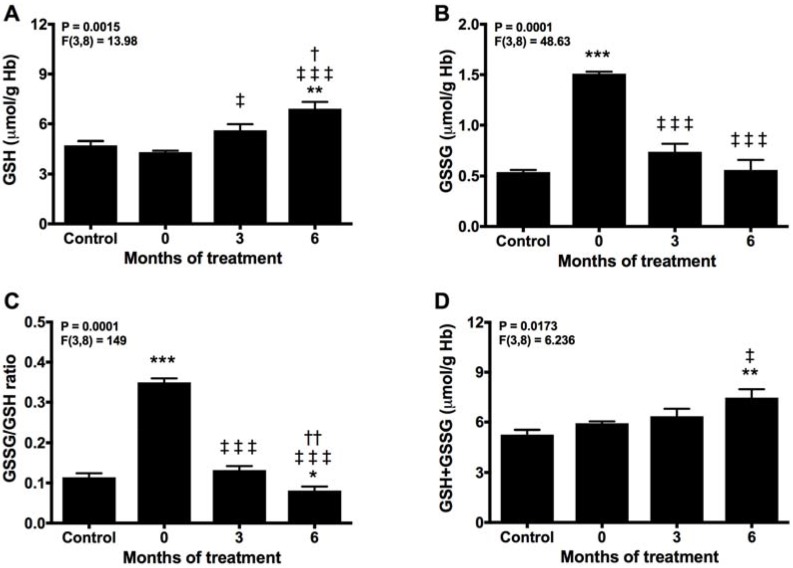
Glutathione turnover in erythrocytes before (0) and after 3 and 6 months of treatment with melatonin, compared with healthy controls. (**A**) GSH, (**B**) GSSG, (**C**) GSSG/GSH ratio, and (**D**) total glutathione. * *p* < 0.05, ** *p* < 0.01 and *** *p* < 0.001 vs. control; ‡ *p* < 0.05, and ‡‡‡ *p* < 0.001 vs. 0; † *p* < 0.05, and †† *p* < 0.01 vs. 3.

Due to its intracellular millimolar concentration, GSH constitutes a major determinant of redox defense [36]. Glutathione is used by either GST and GPx to detoxify and to reduce oxidative damage. In these reactions, GSH is consumed and oxidized to GSSG. Hence, we found a significant reduction in GSH and a parallel rise in GSSG levels in the patients compared with controls before treatment (Figure 2A,B). These changes are reflected in the enhanced GSSG/GSH ratio, suggestive of an elevated oxidative stress (Figure 2C). This condition was counteracted after 3 months of melatonin therapy, which recovered GSH levels even above the controls and reduced that of GSSG, turning the GSSG/GSH ratio towards a reduced condition. During the following 3 months of treatment with melatonin, the GSSG/GSH ratio further decreased, due to the elevation of GSH and decay in GSSG levels. Moreover, total glutathione levels rose with melatonin treatment (Figure 2D), suggesting de novo synthesis of GSH probably related to the effect of melatonin to enhance the expression and activity of γ-glutamylcysteine synthase [37].

**Figure 3:**
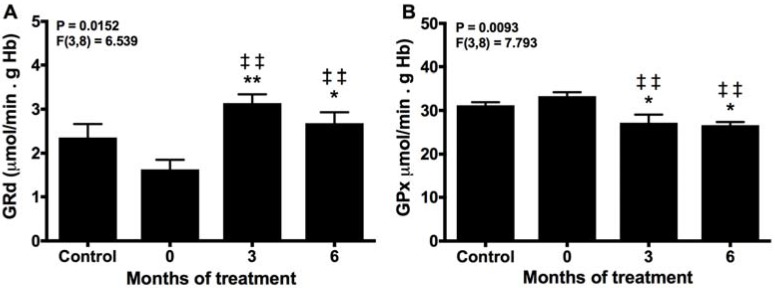
Erythrocyte activities of GRd (**A**) and GPx (**B**) before (0) and after 3 and 6 months of treatment with melatonin, compared with healthy controls. * *p* < 0.05, and ** *p* < 0.01 vs. control; ‡‡ *p* < 0.05 vs. 0.

The GSSG/GSH ratio is controlled by GPx and GRd in the so-called glutathione cycle; GPx consumes GSH to eliminate peroxides, yielding GSSG that in turn should be reduced to GSH again to support the activity of GPx. The reduction of GRd activity in untreated patients supports the excessive consumption of GSH with the formation of GSSG (Figure 3A). This also explains why the activity of GPx remained unchanged in these patients (Figure 3B). The activity of GRd was augmented significantly at 3 and 6 months of treatment, and their effects were also accompanied by a reduction in GPx activity, maintaining the GSH pool and reducing the oxidative stress.

### 2.3. Melatonin Counteracts Proinflammatory Cytokines

**Figure 4:**
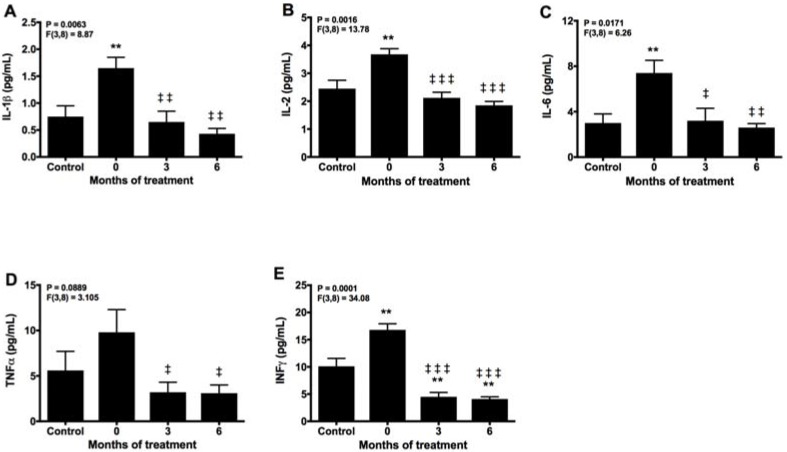
Plasma levels of IL-1β (**A**), IL-2 (**B**), IL-6 (**C**), TNFα (**D**), and INFγ (**E**) and after 3 and 6 months of treatment with melatonin, compared with healthy controls. ** *p* < 0.01 vs. control; ‡ *p* < 0.05, ‡‡ *p* < 0.01, and ‡‡‡ *p* <0.001 vs. 0.

Inflammation is the other important manifestation in CMT patients. Before melatonin treatment, significant elevation of all pro-inflammatory cytokines was detected compared with controls (Figure 4A–E). The levels of IL-1β, IL-2, IL-6, and TNFα were reduced to control values at 3 months of melatonin treatment, whereas INFγ decreased below controls after 3 months of therapy with melatonin. Moreover, IL-1β, IL-2, and IL-6 were further reduced at the end of the study.

### 2.4. Melatonin Neutralizes Markers of Oxidative Damage and Inflammation

**Figure 5:**
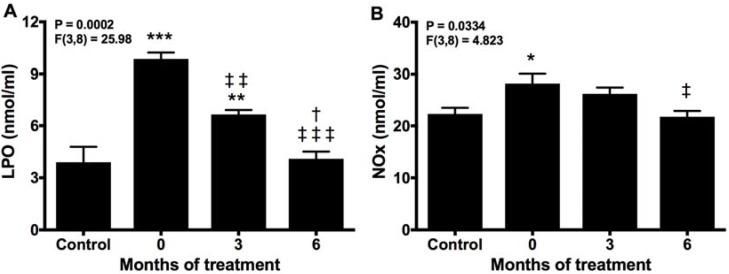
Plasma products of lipid peroxidation (LPO) (**A**) and NOx (**B**) before (0) and after 3 and 6 months of treatment with melatonin, compared with healthy controls. * *p* < 0.05, ** *p* < 0.01, and *** *p* < 0.001 vs. control; ‡ *p* < 0.05, ‡‡ *p* < 0.01, and ‡‡‡ *p* < 0.001 vs. 0; † *p* < 0.05 vs. 3.

High SOD and GST and low GRd activities reflect a hyperoxidative condition that was assessed by analyzing lipid peroxidation and nitrite production. Both of these, LPO and NOx, especially the former, were significantly elevated in untreated patients compared with controls. Melatonin reduced both parameters after 3 months of treatment, and returned LPO and NOx to control values after 6 months of treatment with melatonin (Figure 5A,B).

### 2.5. Effects of Melatonin on Biochemical Markers

Table 1 shows the effects of treatment with melatonin on the plasma markers analyzed. CPK, LDH, GOT, GPT, aldolase, and alkaline phosphatase, which were elevated over the control values before treatment, were reduced significantly after 6 months of melatonin therapy. Of note, CPK decreased by 43%; LDH by 17%; AST, 20%; ALT, 31%; aldolase, 49%, and alkaline phosphatase by 19%.

## 3. Discussion

The results of this study provide the first evidence to support the beneficial effect of melatonin in CMT1 patients. Our results show an increased oxidative stress and inflammation in erythrocytes and plasma of CMT1 patients; these findings were likewise related to the oxidative damage present in other tissues [31,38], and they may reflect the inflammatory demyelinating process [39]. Collectively, the results reported here support the existence of a hyperoxidative state in CMT1 patients. Importantly, treatment with melatonin for 6 months returned these markers to the same basal oxidative and inflammatory condition present in healthy children. The dose of melatonin used, i.e., 10 mg at morning and 60 mg at night, was chosen from a clinical trial in Duchenne muscular dystrophy patients who were significantly improved after melatonin administration [31,32]. Our results indicate that oxidative stress and inflammation are pathogenically important in CMT1, and suggest a potential benefit of melatonin in these patients.

CMT1A disease commonly appears in the second decade of life, with lower limb effects that extend to upper limbs as the disease progresses. CMT1A patients have the PMP22 duplication and, although subacute, acute, and chronic inflammatory demyelinating polyneuropathy forms have been described [40], the patients display typical phenotype of neuropathy, with reduced nerve conduction velocity and demyelination [14,41]. Axonal degeneration and increased disability occur over time. Among other molecular mechanisms of demyelination in CMT1, oxidative stress, inflammation, autophagy impairment, and mitochondrial dysfunction, have been proposed [7,8,9,13,14,15,16,42,43]. Moreover, skeletal muscle atrophy induced by denervation is associated with excessive mitochondrial ROS formation [44].

To provide insights into the role of free radicals and neuroinflammation in CMT1A pathophysiology, we measured the first and second steps of the endogenous antioxidative defense including SOD and GST activities, and the GSH cycle. For this study, we used erythrocytes of healthy subjects and CMT1A patients since the former do not exhibit to oxidative damage [45]. Our results demonstrated an enhanced activity of SOD in the patients, compared with controls, which indicates an overproduction of superoxide anion (O_2_^•−^) the oxygen radical generated by the one electron reduction of oxygen; this radical forms mostly in the mitochondria [46]. Since O_2_^•−^ is converted to H_2_O_2_, which easily passes through cell membranes, the oxidative damage is spread to other cells including erythrocytes where high SOD activity reflected a hyperoxidative status. SOD was decreased by melatonin treatment even below the control values, which reflects a reduction of oxidative stress in the CMTA1 patients.

GST is another enzyme responsible for radical detoxification, redox homeostasis, and S-glutathionylation of target proteins [47]. Under normal conditions GST, along with other antioxidant enzymes, such as SOD and GPx, provide the cell with protection against a range of harmful electrophiles produced during oxidative damage [48]. The GST domain of ganglioside-induced differentiation-associated protein 1 (GDAP1), a mitochondrial fission factor which lead to recessive or dominantly inherited CMTs, is involved, with GST, in the control of GSH content especially at the mitochondrial level [13,49]. Several reports link mutations in GDAP1 with oxidative stress in CMTs due to GSH depletion 46. Here, we show that GST is working properly and its activity is enhanced in CMT1A patients, supporting GSH consumption. In a comparable manner to SOD, GST was significantly reduced by melatonin, consistent with the reduction in the oxidative damage.

The increased activities of both GST and SOD in erythrocytes suggest the existence of an elevated redox status in CMT1A, which may cause the compensatory elevation of these enzymes. SOD activity yields huge amounts of hydrogen peroxide (H_2_O_2_), which can be detoxified by GPx; in this process, GSH is consumed. Together with GST, which also consumes GSH, the GSH pool can be drastically reduced, jeopardizing the endogenous antioxidant defense. Our study is consistent with the observation that the levels of GSSG and the GSSG/GSH ratio are higher in patients than in healthy subjects, coinciding with a decline in GSH [32]. Under these conditions, the endogenous antioxidative defense capacity is weakened in CMT1A patients. The elevated GSSG/GSH ratio here reported in these patients is consistent with an elevated intracellular hyperoxidative status [35]. GSH can also act as a direct free radical scavenger through electron donation [50]. The GSSG/GSH ratio also reflects the state of the enzymes of the glutathione cycle, GPx and GRd, which play a major role detoxifying harmful peroxides [51]. GPx transforms H_2_O_2_ to water and in this reaction, oxidizes GSH to GSSG; GRd, in turn, recycles GSSG back to GSH, supporting the activity of GPx. The finding that CMT1A patients have a reduced activity of GRd explains the diminished GSH levels and are similar to previously published observations [13]. Thus, the detoxifying efficiency of GST and GPx is impaired by the reduced GSH availability in CMT1A patients and, thus, ROS remain elevated.

Accumulation of lipid hydroperoxides is a consequence of ROS. When lipid breakdown occurs in cell membranes, their structure and function is altered which can lead to cell death [52]. ROS initiate an autocatalytic process of LPO, producing large amounts of toxic compounds, including 4-hydroxynonenals, which are an index of the peroxidation of membrane lipids [53,54]. These features of lipid damage were found in the CMTA1 patients, with a significant increase of LPO in parallel with the reduction of the antioxidant status. In experimental neuropathy, similar changes in antioxidant defense and LPO in blood and in nerve tissues of rats were reported; thus, our observations on the blood of patients is a valuable index of the oxidative damage to nerves [15]. Moreover, it has been shown that GST can detoxify LPO conjugating 4-hydroxynonenal, a product of LPO, with GSH, reducing membrane damage [55]. Our findings, however, do not support a significant effect of GST to reduce LPO, probably because the excess of ROS exceeded the capacity of GST detoxification in CMT1A patients. Melatonin is highly efficient in reducing LPO under different pathophysiological conditions, preserving the membrane fluidity [56,57]. After the administration of melatonin to CMT1A patients, it reduced LPO levels to control values after 6 months of treatment, and also reduced GST activity. These data, with the recovery of the GSH pool by melatonin, further support that a major effect of melatonin is to inhibit ROS-mediated damage, diminishing the requirements of the endogenous antioxidant system defense.

ROS, and LPO particularly, represent important triggers of NF-κB activation, resulting in an inflammatory cascade [58]. Here, the increase in the proinflammatory cytokines found in our study reflects the activation of the NF-κB pathway and, thus, supports the evidence suggesting that inflammatory processes contributed substantially to the neuropathy process [11,59,60]. These findings are supported by the elevated levels of NOx in CMT1A patients; NOx reflects the induction of iNOS by NF-κB. The inhibitory properties of melatonin on the NF-κB pathway of the innate immunity [20] observed here involve the reduction of proinflammatory cytokines and NOx. Importantly, IL-1β is produced by the caspase-1 cleavage of a pro-caspase-1, transcriptionally controlled by NF-κB. But caspase-1 is in turn activated by the NLRP3 inflammasome, the other pathway of the innate immunity that is also blocked by melatonin [23,61,62]. Thus, the inhibition of IL-1β by melatonin reported here suggests that the neuroinflammatory process in CMT1A neuropathy involves both NF-κB and NLRP3 inflammasome [20]. TNF-α can also stimulate the production of mitochondrial ROS, thus creating a positive feedback loop by which the increased ROS cause further activation of the inflammatory pathways; this links CMT1A neuropathy to mitochondrial impairment. In recent years, mutations of genes encoding proteins that play important roles in mitochondrial dynamics have been identified in CMTs [63], and relationships between mitochondrial bioenergetic changes in CMTs have been also observed [13,49,64]. Thus, exploring mitochondrial function in CMTA1 may yield important clues to the pathogenicity of CMT1A and in the therapeutic benefits of melatonin [25].

It should be noted here that there are two main limitations of our study. The first one is the small number of patients studied. Because it is a rare disease, a larger number of CMT1 patients is difficult to collect for the study, but we are now attempting to do a new study with the same dose of melatonin and more time of treatment in more patients. The second limitation of the study is the lack of clinical data at this moment. Together with the pediatric unit, we decided on the dose of melatonin used in the treatment of the children according to previous studies [32]; then, the blood samples were obtained and analyzed in our lab, but we had no access to the clinical data. The new study that we are promoting is devoted to increasing not only the number but also the clinical data of the patients enrolled in the study, to further support the beneficial effects of melatonin therapy reported here.

Overall, our results indicate a significant oxidative stress and neuroinflammation in CMT1A patients, which may participate in the pathogeny of the disease. Our data also suggest more complex pathogenic mechanisms, involving, besides the NF-κB pathway, mitochondrial impairment and ROS production, which are triggers for the NLRP3 inflammasome activation. The existence of oxidative stress in CMT1A led to the use of antioxidants such as vitamin C in several clinical trials [65]. The lack of benefits of vitamin C and other antioxidant vitamins has been reported; the lack of efficacy of vitamin antioxidants may be due to (a) their pro-oxidant properties which enhance mortality [66,67], and (b), their lack of effects at the mitochondrial level [26]. Melatonin, however, is a different class of antioxidant and anti-inflammatory molecule that can reach all cellular compartments acting in situ to recover homeostasis [24]. Additionally, melatonin has been proved to have analgesic properties and it relieves neuropathic pain by acting on its MT2 membrane receptors [68,69]. Thus, melatonin should be seriously considered as a useful therapy in CMT1A pathology due to its multiple-drug properties.

## 4. Materials and Methods

### 4.1. Patients

The study was carried out on 3 pediatric patients, two boys and one girl, aged 8–10 years, who were followed in the Neuropediatric Unit of the Granada University Hospital (Granada, Spain). These patients were diagnosed for Charcot-Marie-Tooth (CMT) type 1A. Melatonin (Fagrón Ibérica, Barcelona, Spain) therapy consisted in the administration of two oral daily doses of melatonin capsules for 6 months, one at 09:00 h (10 mg melatonin) and the other at 21:00 h (60 mg melatonin), respectively. The daily schedule of melatonin administration was chosen to maintain the night/day blood melatonin concentration difference. A group of 8 healthy age- and sex-matched (4 boys and 4 girls, aged 7–11 years) subjects was used as a control group (C).

### 4.2. Samples

Blood samples were obtained from the antecubital vein in all patients at 09:00 h before starting with melatonin therapy, and 3 and 6 months during melatonin treatment. Samples were centrifuged at 3000 *g* for 10 min and erythrocytes were separated, washed twice with cold saline, and frozen at −80 °C until the biochemical assays were performed. On the day of the experiment, washed erythrocytes were hemolized in phosphate buffer (10 mM sodium phosphate, 1 mM EDTA-Na2, pH 6.25, deproteinized with ice-cold 10% trichloroacetic acid, and centrifuged at 20,000 *g* for 15 min. Supernatants were then used for the measurements.

### 4.3. Superoxide Dismutase Assay

SOD activity was measured in hemolized erythrocytes following the method of Misra and Fridovich [70]. This method is based on the inhibition of adrenaline autooxidation in alkaline medium by the enzyme. Briefly, an aliquot of hemolized erythrocytes was incubated in 50 mM Na_2_CO_3_ buffer containing 0.1 mM EDTA, pH 10.2 and 200 μL of a solution of 6 mM epinephrine in 1 mL HCl. The reaction was followed a 480 nm by 10 min in a spectrophotometer (Shimadzu Deutschland GmBH, Duisburg, Germany). SOD activity was expressed in adrenaline units (U/g Hb).

### 4.4. Glutathione S-Transferase Assay

Washed erythrocytes were hemolized in 1:20 phosphate buffer (10 mM sodium phosphate, 1 mM EDTA-Na2, pH 6.25. 5 mL of hemolisate were mixed with 5 mL Sephadex CM-50 with the same buffer (1:100); vortex for 3 min, and centrifuged at 6500 *g* for 10 min. One aliquot of the supernatant was maintained at 4 °C to calculate the total GST activity, following the procedure of Habig et al. [71], using 30 mM 1-chloro-2,4-dinitrobenzene as substrate. The activities were spectrophotometrically measured for 3 min at 340 nm, and expressed as μmoL/min∙g Hb.

### 4.5. GSH and GSSG Determinations

For glutathione measurement, 10 μL of the supernatant of hemolyzed erythrocytes were incubated with 10 μL of ethanol ophthalaldehyde solution (1 g/L) and 180 μL phosphate buffer A (100 mM sodium phosphate, 5 mM EDTA-Na2, pH 8.0) for 20 min at room temperature. Then, the fluorescence of the samples was measured at 350 nm excitation and 420 nm emissions in a plate-reader spectrofluorometer (Bio-Tek Instruments, Inc., Winooski, VT, USA) [72]. For glutathione disulfide measurement, 200 μL aliquots of supernatants were preincubated with 80 μL N-ethylmaleimide solution (5 g/L in distilled water) for 40 min at room temperature and then alkalinized with 720 μL sodium hydroxide 0.1 N. Aliquots of 30 μL were then incubated with 10 μL ophthalaldehyde solution and 160 μL sodium hydroxide 0.1 N for 25 min at room temperature, and the fluorescence was then measured [25]. Glutathione concentrations were calculated according to standard curves previously prepared. Levels are expressed in μmoL/g Hb.

### 4.6. Glutathione Peroxidase and Reductase Assays

To measure glutathione peroxidase (GPx) activity, 10 μL of each supernatant from hemolized erythrocytes were added to 240 μL of working solution containing buffer A plus 4 mM sodium azide, 60 mM glutathione, 20 mM NADPH, and 0.5 U/L glutathione reductase. After incubation for 5 min at room temperature, the reaction was started by adding 10 μL of 0.3% cumene hydroperoxide and the GPx activity was determined following the oxidation of the NADPH for 3 min at 340 nm in an UV spectrophotometer (Shimadzu Deutschland GmBH, Duisburg, Germany). To measure glutathione reductase (GRd) activity, 35 μL of each supernatant were added to 465 μL of working solution containing buffer A plus 2.5 mM disulfide glutathione. After incubation for 5 min at room temperature, the reaction was started by adding 8.5 μL of 12 mM NADPH and the GRd activity was spectrophotometrically determined for 3 min at 340 nm [73]. GPx and GRd activities are expressed as nmol/min∙g Hb. In both cases, non-enzymatic NADPH oxidation was subtracted from the overall rates.

### 4.7. Plasma Cytokines Determination

The Milliplex Human Cytokine immunoassay kit (Millipore Corp., Billerica, MD, USA) was used to profile expression of five inflammatory mediators (IL-1b, IL-2, IL-6, TNF-α, and INF-γ). The assay was performed according to the manufacturer’s instructions. Briefly, 50 µL of working solution containing multiple microbeads labeled with specific antibodies against each of the aforementioned cytokines were added into each well, washed twice with 200 µL of Linco-Plex wash buffer and filtered to dryness. Then 25 µL thawed plasma aliquots diluted 1:4 with the specific Linco-Plex sample diluents were added to each well and incubated for 60 min at room temperature. After a wash step (twice) with 200 µL Linco-Plex wash buffer, the beads were incubated with 25 µL of the detection antibody cocktail for 30 min at room temperature, each antibody specific to a single cytokine. After other twice wash steps with 200 µL Linco-Plex wash buffer, the beads were incubated with 25 µL of the streptavidin-phycoerythrin solution for 30 min at room temperature and washed twice again. The beads were resuspended in each well with 100 µL of the Linco-Plex Sheath fluid and the concentration of each cytokine was determined using the array reader. A parallel standard curve was constructed for each cytokine. Levels are expressed in ng/L.

### 4.8. Lipid Peroxidation Assay

Plasma samples were thawed and centrifuged at 5000 *g* for 5 min, and 200 µL of the supernatants were used for LPO measurements. For this purpose, a commercial LPO assay kit that estimated both malondialdhehyde (MDA) and 4-hydroxyalkenals (4HDA) was used (Bioxytech LPO- 568 assay kit; OxisResearch, Portland, OR, USA) [53]. LPO concentrations are expressed in µmol/L.

### 4.9. Nitrite Plus Nitrate Determination

Thawed plasma samples were deproteinized with ice-cold 6% sulfosalicylic acid, incubated at room temperature for 30 min and centrifuged at 10,000 *g* for 15 min; then, 50 µL supernatant were incubated with 4 µL 1.25% NaOH, 36 µL of 14 mM phosphate dehydrogenase, 750 µM glucose-6- phosphate, 30 mU nitrate reductase, and 10 µL 3 µM NADPH for 60 min at room temperature. The concentration of nitrites was measured following the Griess reaction which coverts nitrite into a colored azo compound spectrophotometrically detected at 550 nm [74]. Plasma levels of NOx are expressed in µmol/L.

### 4.10. Biochemical Analysis

Patient samples were analyzed within 24 h by the clinical analysis laboratory of the University Hospital of Granada. The following markers were measured for muscle injury: creatine kinase (CK), lactate dehydrogenase (LDH), aspartate aminotransferase (AST), alanine aminotransferase (ALT), gamma-glutamyltransferase (γGT), aldolase, alkaline phosphatase, myoglobin, calcium, and phosphate.

### 4.11. Statistical Analysis

Data are expressed as the mean (SEM). ANOVA followed by Fisher’s LSD post hoc test were used to compare the means between groups (before and after the treatment). A *p* value of less than 0.05 was considered statistically significant.

### 4.12. Ethical Approval

Informed consent was obtained from all subjects and from the Hospitals Ethical Committee, (no. 0191/06) according to the 1983 revised Helsinki Declaration of 1975.

## 5. Conclusions

Charcot-Marie-Tooth (CMT) neuropathy is a hereditary motor and sensory neuropathy. Specifically, CMT type 1 courses with reduced nerve conduction velocities and hypertrophic demyelination. These clinical features parallel with inflammation and oxidative stress, both of which aggravate the progression of the disease. The clinical experience with melatonin in reducing oxidative damage and counteracting inflammation led us to its therapeutical use in CMT1 patients. After melatonin administration, markers of oxidative damage, antioxidative defense, and inflammation, were significantly reduced at 3 months of treatment, and fully counteracted at 6 months of treatment. Together with the reduction of biochemical markers of skeletal muscle and liver damage, our results support the interest of the clinical therapy with melatonin in the treatment of CMT1 disease.

## Figures and Tables

**Table 1:** Plasma biochemical markers analyzed.

Variable	Control	Melatonin Treatment (Months)		
		0	3	6
CPK	114.3 ± 45.2	146.3 ± 73.2	155.5 ± 70.5	83.7 ± 40.8 ***^,###^
LDH	248.4 ± 44.6	407.2 ± 79.2 **	380.4 ± 46.2 *	339.1 ± 17.5 *^,#^
AST	23.4 ± 5.1	29.7 ± 5.3 *	25.6 ± 5.3	23.8 ± 6.0 ^#^
ALT	24.4 ± 3.2	17.7 ± 0.6 *	14.8 ± 3.0 **	12.2 ± 2.1 ***
γGT	25.5 ± 2.5	11.9 ± 5.5 **	13.2 ± 2.5 **	16.3 ± 8.9 **^,#^
Aldolase	6.7 ± 2.1	4.7 ± 1.6	3.5 ± 0.7 **	2.4 ± 0.2 ***^,#^
Alkaline phosphatase	<350	188.5 ± 101.2	174.2 ± 75.0	153.2 ± 75.8
Myoglobin	50.6 ± 10.4	23.7 ± 2.5 ***	22.9 ± 3.1 ***	24.7 ± 2.4 ***
Calcium	9.7 ± 0.5	9.3 ± 0.1	9.5 ± 0.3	9.4 ± 0.3
Phosphate	3.8 ± 0.8	4.3 ± 0.1	4.7 ± 0.1	4.4 ± 0.1

Data are expressed as the means ± SEM. Enzyme activities are expressed in U/L; myoglobin concentration in ng/L, and calcium and phosphate levels in mg/dL. * *p* < 0.05, ** *p* < 0.01, and *** *p* < 0.001 vs. control; # *p* < 0.05, and ### *p* < 0.001 vs. 0.
